# BUILDING THE RESEARCH PRACTICE PARTNERSHIP TO PROMOTE CONTINUOUS QUALITY IMPROVEMENT IN ADULT PROTECTIVE SERVICES

**DOI:** 10.1093/geroni/igad104.1905

**Published:** 2023-12-21

**Authors:** Joan Davitt, Todd Becker, Jocelyn Brown, Erin Barry-Dutro, Greg Sesek, Peiyuan Zhang

**Affiliations:** University of Maryland, Baltimore, Baltimore, Maryland, United States; University of Maryland, Baltimore, Baltimore, Maryland, United States; University of Maryland, Baltimore, Baltimore, Maryland, United States; University of Maryland, Baltimore, Baltimore, Maryland, United States; Department of Human Services, Baltimore, Maryland, United States; University of Maryland, Baltimore, Baltimore, Maryland, United States

## Abstract

Maryland’s Department of Human Services, Office of Adult Services developed a multiyear plan to strengthen Adult Protective Services. This plan included the development of a partnership between OAS and the University of Maryland School of Social Work to develop, implement and sustain a Continuous Quality Improvement (CQI) review process for Adult Services Programs (APS) to improve outcomes for vulnerable, elderly, and disabled adults. This paper focuses on partnership development between state government and university researchers to engage collaboratively in continuous quality improvement and intervention efficacy evaluation. Challenges and strategies for building logic models, developing performance measurement strategies, translating existing administrative data into research ready formats, and engaging stakeholders at the state level and in local departments of social services will be discussed. Key challenges included, staffing issues, technology, access to administrative data, and inadequate system resources to engage in research. Strategies for overcoming these challenges will be discussed.

